# Tumour Promotion by the Neutral Fraction of Cigarette Smoke

**DOI:** 10.1038/bjc.1960.72

**Published:** 1960-12

**Authors:** G. R. Clemo, E. W. Miller


					
651

TUMOUR PROMOTION BY THE NEUTRAL FRACTION

OF CIGARETTE SMOKE

G. R. CLEMO AND E. W. MILLER

From The Laboratory, Cherryburn, Mickley-on-Tyne, and Cancer Research Laboratory,

Department of Pathology, Royal Victoria Infirmary, Newcastle upon Tyne

Received for publication October 8, 1960

IT is known that the incidence of lung cancer amongst heavy cigarette smokers
is higher in urban than in rural districts. While this might be attributed solely
to exposure to the carcinogenic substances present in the urban atmosphere (e.g.
particularly smoke from coal fires, also diesel fumes and car exhaust fumes
(Kotin, 1956)), there is also the possibility of a co-carcinogenic action between
cigarette smoke and city smoke. The following series of experiments was
designed to test this hypothesis. Since the experiments were begun three years
ago, Gellhorn (1958), Roe, Salaman and Cohen (1959) and Wynder and Hoffman
(1960) have confirmed the presence of incomplete carcinogens in cigarette smoke
as foreshadowed by Hamer and Woodhouse (1956) and Gwynn and Salaman
(1956).

MATERIAL AND METHODS

One hundred and fifty-six C57BL mice (68 females, 88 males) were divided
into 7 groups for the purposes of treatment. Aged between 3 and 6 weeks at the
beginning of the experiment, the mice were allowed to live to the end of their
normal lives or, in the case of those with skin tumours, they were killed when
the tumours became large or frankly malignant. All skin tumours except the
smallest papillomata were sectioned and examined histologically. A tumour
was judged to be malignant when the panniculus carnosus was invaded. Tumours
were classed as "probably malignant "when the tumour cells had not yet reached
the muscle although other signs of malignancy were present.

The test substances in solution were applied with two strokes of a No. 4 paint-
brush to the skin in the interscapular region. There were three substances:
fraction " C " from city smoke, a known carcinogenic material (Clemo, Miller and
Pybus, 1955), applied as a 1-0 per cent solution in benzene; croton oil, a known
tumour promoter, applied as a 0.5 per cent solution in acetone; and the neutral
fraction of cigarette smoke applied as a 10 per cent solution in benzene. This
last fraction was extracted from the whole tar from cigarette smoke as described
by Clemo (1958) and approximately 6.4 mg. was applied to each mouse at each
painting.

The 7 groups in the experiment received the following treatments:

Group I.-Twenty-one mice (9 females, 12 males) were painted 3 times a week,
for 2 weeks only, with fraction "C ". They received no further treatment.

Group II.-Twenty-one mice (6 females, 15 males) were painted with fraction
C" as in Group I. After an interval of 3 weeks they were painted with neutral
fraction 3 times a week till death.

G. R. CLEMO AND E. W. MILLER

Group III.-Twenty-four mice (15 females, 9 males) were painted with fraction
"C" as in Group I. After an interval of 3 weeks they were painted with croton
oil twice weekly until death.

Group IV.-Twenty-two mice (8 females, 14 males) were painted 3 times a
week, for 2 weeks only, with neutral fraction. They received no further treatment.

Group V.-Twenty-one mice (6 females, 15 males) were painted with neutral
fraction as in Group IV. After an interval of 3 weeks they were painted with
fraction " C " 3 times a week until death.

Group VI.-Twenty-five mice (11 females, 14 males) were painted with neutral
fraction as in Group IV. After an interval of 3 weeks, they were painted with
croton oil twice weekly until death.

Group VII.-Twenty-three mice (13 females, 10 males) were painted with
croton oil twice weekly until death.

RESULTS

The results are summarised in Table I.

Group I.-The dose and duration of painting with fraction " C " were arbit-
rary. It was known to produce many skin tumours in C57BL mice when applied
throughout life (Clemo and Miller, 1957). From the present experiment it was
evident that a minimal carcinogenic dose had been given. Two males (aged 19
and 29-5 months) developed 3 small papillomata, 2 of which, at the site of painting,
were superficial, while in the third (slightly larger and on the abdominal surfacc)
the epithelium had not yet reached the panniculus carnosus. One female deve-
loped a rapidly growing spindle-celled subcutaneous sarcoma over the left scapula,
at the age of 20 months. The non-tumour mice all lived well into tumour age,
4 dying with leukaemia, 2 with hepatomata and one with leukaemia plus hepa-
toma.

Group II.-Three males developed epitheliomata (one per mouse) at the site
of painting, all growing steadily so that the mice had to be killed 3 to 4 months
after the first appearance of the lesions; 7 papillomata appeared in 5 other males,
all at the site of painting. In 4 females there were 8 skin tumours (one mouse had
4 and in another mouse 2 coalesced to form one); of these, 5 were innocent
papillomata but 3 were classified as "probably malignant ". Non-tumour mice
lived well into tumour age; one female died at 11 months of leukaemia, the other
at 15.5 months of pneumonia, while gross kidney disc:-,se caused the deaths of
the 7 non-tumour males.

Group III.-The maximum total number of skin tumours in the 9 tumour
females was 14, but 4 disappeared before death, leaving a final total of 10, the
greatest individual number being 4; of these 10, one grew slowly but steadily
to become "probably malignant" at the time of death 8 months later, the rest
remaining small. The three tumours in males (one each) were all small. The
non-tumour mice were all of tumour age and the majority died with grossly
diseased kidneys.

Group I V.-In this group 2 male mice each developed one small skin papil-
loma; one of these, in the lumbar region, disappeared before death; the second
was in the centre of the akdominal surface. The remaining mice died at tumour
age without skin tumours but 3 had leukaemia, one a very large lung tumour
(rare in this strain), one had haemangioma of the spleen, one a hepatoma and the
rest had diseased kidneys.

652

TUMOUR PROMOTION BY CIGARETTE SMOKE

653

.-I- - - I --

O C> to (C) O O

00 aq M c ? C,q

aq aq

aq -4 aq -4 P-4

40 LO Lo xo xo 0
<? ?o C? ci) ?- Q'o

aq

aq    N

0 xo 0
aq aq aq

xo
l'O t-,

xo    Lo

L'O   4;

aq aq    aq     -4 cq

0 CD)

r4D
o

cD0

0     ?

? ~-

0 ~~~         o

- 000

Qo Q

e-, -._

;Yn

I"

5 d
o?-

I
t
c

I
c

I
PL

4
4

1
4

4
1
4
1

4
4
4
3
I

c 'd

b,.S
o~-

qD
*cb.

E--t

4-D

0

eb,

b,  ."'

4QD

ca .

O

--

4-'-?-
0

C3
0

bo
.-4
1-4

C3

E -?

4

4
C3
4
0

1?0
P-1

r' &0~~~

0~~~~~

5    -o > -0  ?++ +._.

o  f 7 >.  - o

O E-E3i  .   _

n .   X,,   0-  _  _ q m ca _ <

P. 0- t e m Xm m   c > m
0~~~~

A~~~~

+q + +q - aq P o

+++ t
Ca  _   p
c2~~~~

bX IM 4. :5 +-

bo Ca en 4 0

.6, =1: 4 o5 - t-_

o B     0_      . .

, _--

,-4i

_ oo

0 C>
4z o    0c

v0  c'

CD

f0

0     N

4.5 Oa  5 _

Q .  .....
4- .n ;. 4 ,

o    k --i

P0 0 1E

-

Z  O

G. R. CLEMO AND E. W. MILLER

Group V.-The majority of the mice, otherwise healthy, were killed when their
tumours became large and malignant or "probably malignant ", but two had
kidney disease and died before their tumours had grown to any size. Every
mouse had multiple skin tumours. The tumour incidence in males and females
is given in Table II. Although the males had on an average more tumours per
mouse than the females, the difference was not significant either for the maximum
average numbers (column 5) (d - 1.3, 2 x S.E. = 2.1) or for the final average
numbers (column 6) after tumours had coalesced (d = 1.5, 2 x S.E. - 1.9).
But when the proportions of tumours which became malignant or "probably
malignant" were compared (column 11), the difference between the sexes was
significant (d = 36.5, 2 x S.E. = 28.6). The average age of the females at
tumour appearance was 10.8 months (range = 9-0-12.5 months) and that of
the males was 9.8 months (7.5-11.5 months); the average age of the females at
death was 14.2 months (12.0-17*0 months) and of the males was 14.5 months
(11.5-17.5). Thus although the latent period was longer in the females, the
tumours became malignant more rapidly than in the males, and a greater propor-
tion of the tumours became malignant (and "probably malignant ") in the
females.

Group VI.-The only mice to produce skin tumours were 2 males, each having
one small papilloma, one at the age of 14 months (on the forehead) and the other
at 17 months (interscapular); the latter had disappeared at the time of death 2
months later. The mice in this group, although of tumour age, died sooner than
those of other groups, most of them with diseased kidneys.

Group VII.-There was only one tumour mouse, a female with a skin papil-
loma on the left flank at the age of 21 months. That tumour disappeared before
death 7 weeks later, but meanwhile another papilloma, which had appeared in the
dorsal region a week or two after the first, had developed into a small epithelioma.
The remaining mice died tumour-free at a similar age to those in Group VI and
all but one had grossly diseased kidneys.

DISCUSSION

It is clear from these experiments that, under the conditions of treatment
described, the neutral fraction of cigarette smoke can act as a definite tumour
promoter. Some evidence has already appeared in the literature that whole
tar from cigarette smoke may have tumour-promoting properties when applied
after a known carcinogen such as 3: 4-benzopyrene (Hamer and Woodhouse,
1956; Gwynn and Salaman, 1956); these workers found no proof of tumour-
initiation. Gellhorn (1958) demonstrated convincingly the tumour-promoting
activity of whole tobacco tar after the preliminary application of 3: 4-benzo-
pyrene; he found also that although croton oil promoted a higher incidence of
carcinomata, tobacco tar increased the "conversion rate" of carcinomata from
papillomata compared with croton oil. More recently Roe, Salaman and Cohen
(1959) have shown that the phenolic fraction is a tumour promoter, the initiator
in this case being 9,10-dimethyl-1,2-benzanthracene; they proved also that the
neutral fraction is carcinogenic. Wynder and Hoffman (1960) mention the
tumour-promoting properties of the phenol fraction and of the nicotine-free basic
fraction.

As far as is known, the present series of experiments is the first in which the
initiating substance has been, not one of the well-known carcinogens such as

654

TUMOUR PROMOTION BY CIGARETTE SMOKE

3: 4-benzopyrene, but a mixture (of proved carcinogenic power) obtained direct
from city smoke. Although its components are still not wholly identified, fraction

C " was chosen deliberately in order to simulate more closely the actual con-
ditions of daily life. That it is a strong carcinogen was shown once again by
the mice of Group V, to which it was applied until death and in which the tumour
incidence was 100 per cent; this result was the same as had been obtained in
previous experiments with fraction " C " alone and any hypothetical promoting
effect due to the preliminary treatment with neutral fraction would be quite
obscured by the potency of fraction "C ". That a minimal initiating dose was
given to the mice in Group I was shown by the very low tumour incidence in that
group.

The crucial results are given by Groups II and III; in these, fraction " C

applied in minimal dose as initiator was followed in Group II by neutral fraction
and in Group III by croton oil as promoters. Croton oil is a well-known tumour
promoter; it has also been shown to be a weak carcinogen, producing a number
of papillomata which usually regress when treatment ceases, but Roe (1956)
reported 7 malignant tumours in 20 mice which had been treated for from 55 to
72 weeks. In the present instance, applied alone throughout life (up to 21 months'
treatment), it produced in Group VII only one malignant tumour and one papil-
loma which regressed in 23 mice living over one year.

In Group III 12 of the 24 mice developed a total of 16 skin tumours, of which
4 regressed and only one (which took 6 months to grow and was the only tumour
of any size in that group) became malignant; the final tumour incidence was 33
per cent.

In Group II 12 of the 21 mice produced a total of 18 skin tumours, of which
none regressed (but 2 coalesced as they increased in size) and 6 became malignant
or probably malignant, 5 of them attaining quite a large size in from 2 to 4 months.
Thus although the latent period (see Table I) was much longer with neutral
fraction as promoter, there was a much shorter time between tumour appearance
and death than with croton oil as promoter. While the differences in tumour
incidence are not statistically significant, a comparison of the charts of tumour
growth was most convincing and showed that under the conditions of the experi-
ment a 10 per cent solution of neutral fraction applied three times a week was a
more powerful tumour promoter than a 0.5 per cent solution of croton oil applied
twice weekly.

The present experiments provide less certain evidence of tumour intiation by
neutral fraction; with what was intended to be a minimal dose (Group IV)
papillomata appeared in 2 mice out of 22, but one regressed and the other was far
from the site of painting. When, in Group VI, after this preliminary treatment
with neutral fraction, croton oil was applied as in Group II as a promoter, again
only 2 mice developed skin papillomata, no more than might be expected from the
use of croton oil alone. While this might mean that the original dose was too
small to give tumours, in previous experiments in which a 10 per cent solution
of neutral fraction was applied throughout life to mice of the A and C57BL strains
no skin tumours were observed. On the other hand Roe, Salaman and Cohen
(1959) obtained proof of complete carcinogenesis with neutral fraction when they
produced papillomata and two skin carcinomata in 5 mice living up to 60 weeks
(from 37 survivors at 30 weeks), painting being stopped after 47 weeks; they
applied neutral fraction three times weekly at a dose of 40 mg. at each painting.

655

656                 G. R. CLEMO AND E. W. MILLER

In the present work the dose of neutral fraction, forming about 18 per cent by
weight of the whole original tar (Clemo, 1958), was approximately 6-4 mg. per
mouse at each painting. The neutral fraction used by Roe, Salaman and Cohen
(1959) which apparently contained the ester fraction removed from our neutral
fraction, formed approximately 56 per cent by weight of the original tar. It is
thus not possible to compare strictly the individual doses used by ourselves and
by Roe et al. (1959). Gellhorn (1958) gave approximately 50 to 60 mg. of tar
per week to each mouse our dose of 19.2 mg. neutral fraction per week probably
represents about twice as much neutral fraction as would be present in his dose.
Although Gellhorn used the same strength of croton oil, he was giving three times
as much per week as in our experiments; he obtained far more tumours with
croton oil thcan he did with the tar (as promoters), and given the differences in
dosage the present results are not inconsistent with his.

It was noticed in the present experiments that mice receiving croton oil
throughout their lives (Groups III, VI and VII) died much earlier than those in
other groups (except Group V where they all developed tumours) and the majority
had severe kidney disease.

SUMMARY

Experiments are described in which a definite tumour-promoting effect was
observed when the neutral fraction from cigarette smoke was applied to C57BL
mice after an initiating dose of fraction " C" from city smoke. Eighteen skin
tumours, of which 6 became malignant or "probably malignant" were produced
in 12 out of 21 mice painted in the interscapular region throughout their lives
(up to 28 months). Similar treatment with croton oil after fraction " C "resulted
in 16 skin tumours, of which 4 regressed and one became malignant, in 12 out of
24 mice.

There was little evidence of either a tumour-initiating or a complete carcino-
genic effect with a small dose of neutral fraction. No more tumours were pro-
duced with croton oil applied after neutral fraction than were produced by croton
oil alone.

When fraction " C " was applied throughout life, the latent period was longer
in females than in males, but tumour growth was more rapid in the females and a
significantly greater percentage of tumours in females developed to malignancy.

This work was carried out with the aid of a research grant from the North
of England Council of the British Empire Cancer Campaign, for which the authors
would express their gratitude. Thanks are due also to Mrs. Eileen Moody of the
technical staff for her assistance.

REFERENCES
CLEMO, G. R.-(1958) Tetrahedron, 3, 168.

Idem AND MILLER, E. W.-(1957) Brit. J. Catcer, 11, 403.
Iidem AND PYBtS, F. C.-(1955) Ibid., 9, 137.
GELLHORN, A.-(1958) Cancer Res., 18, 510.

GWYNN, R. H. AND SALAMAN, M. H.-(1956) Rep. Brit. Emp. Cancer Campqn., 34, 193.
HAMER, D. AND WooDHoUsE, D. L.-(1956) Brit. J. Cancer, 10, 193.
KOTIN, P.-(1956) Cancer Res., 16, 375.

ROE, F. J. C.-(1956) Brit. J. Cancer, 10, 72.

Idem, SALAMAN, M. H. AND COHEN, J.-(1959) Ibid., 13, 623.

WYNDER, E. L. AND HOFFMAN, D.-(1960) Proc. Amer. Ass. Cancer Res., 3, 164.

				


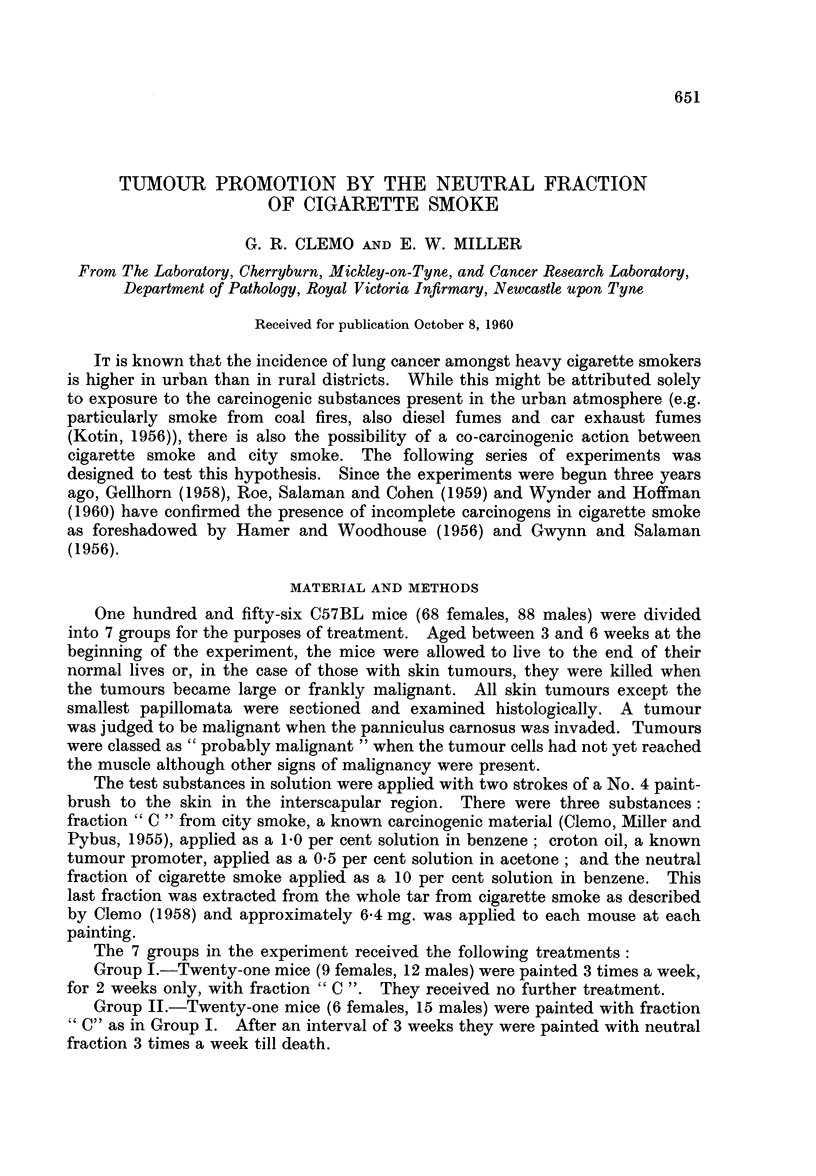

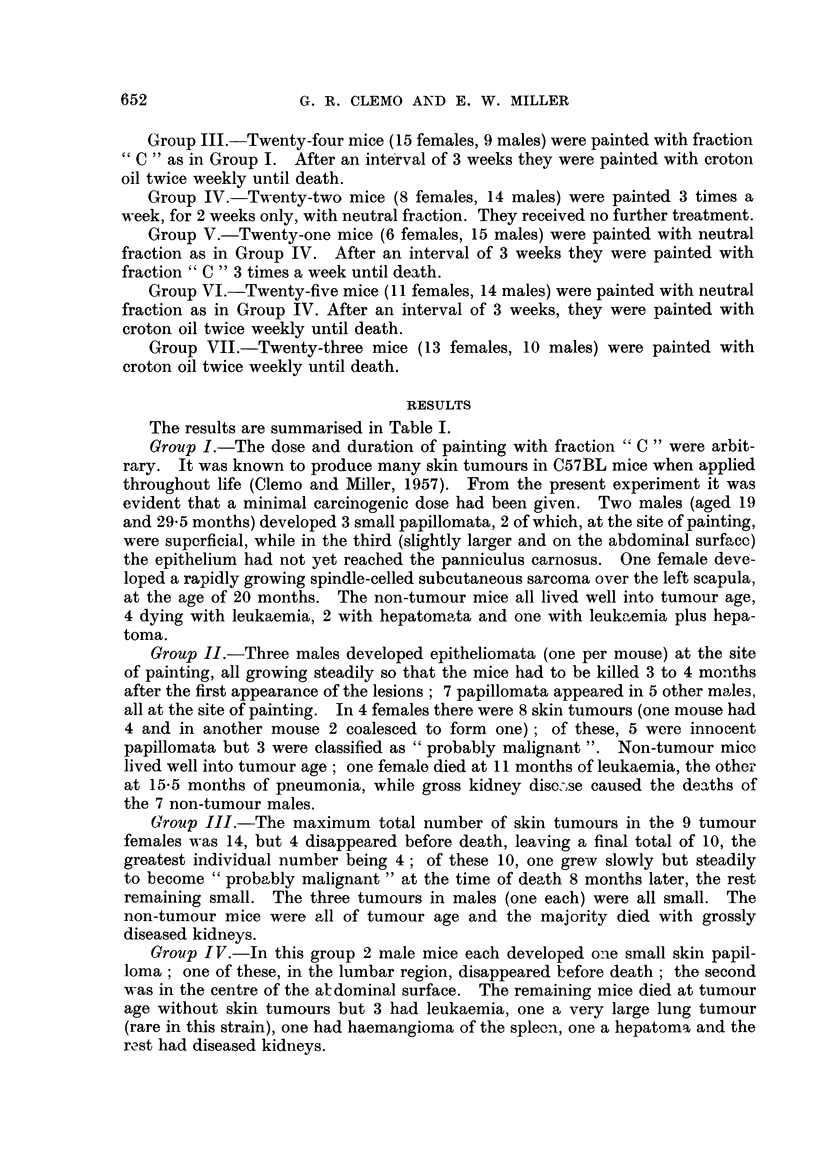

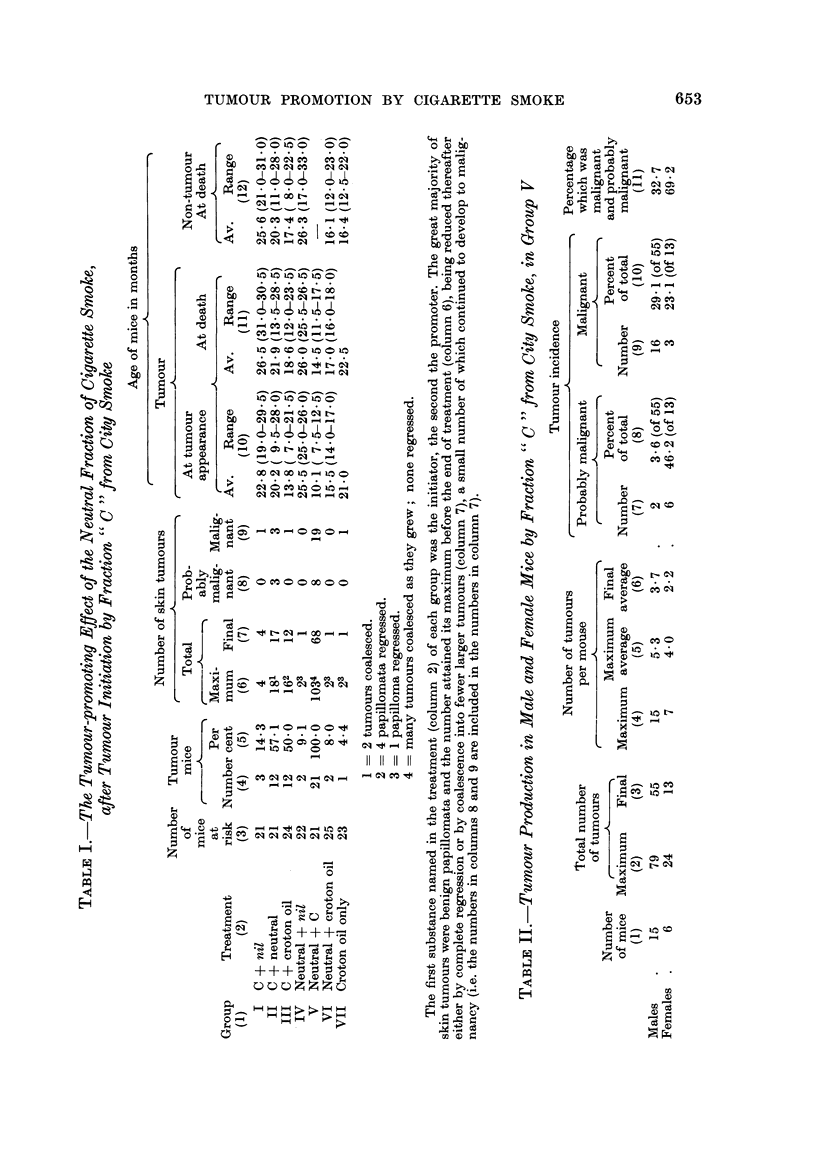

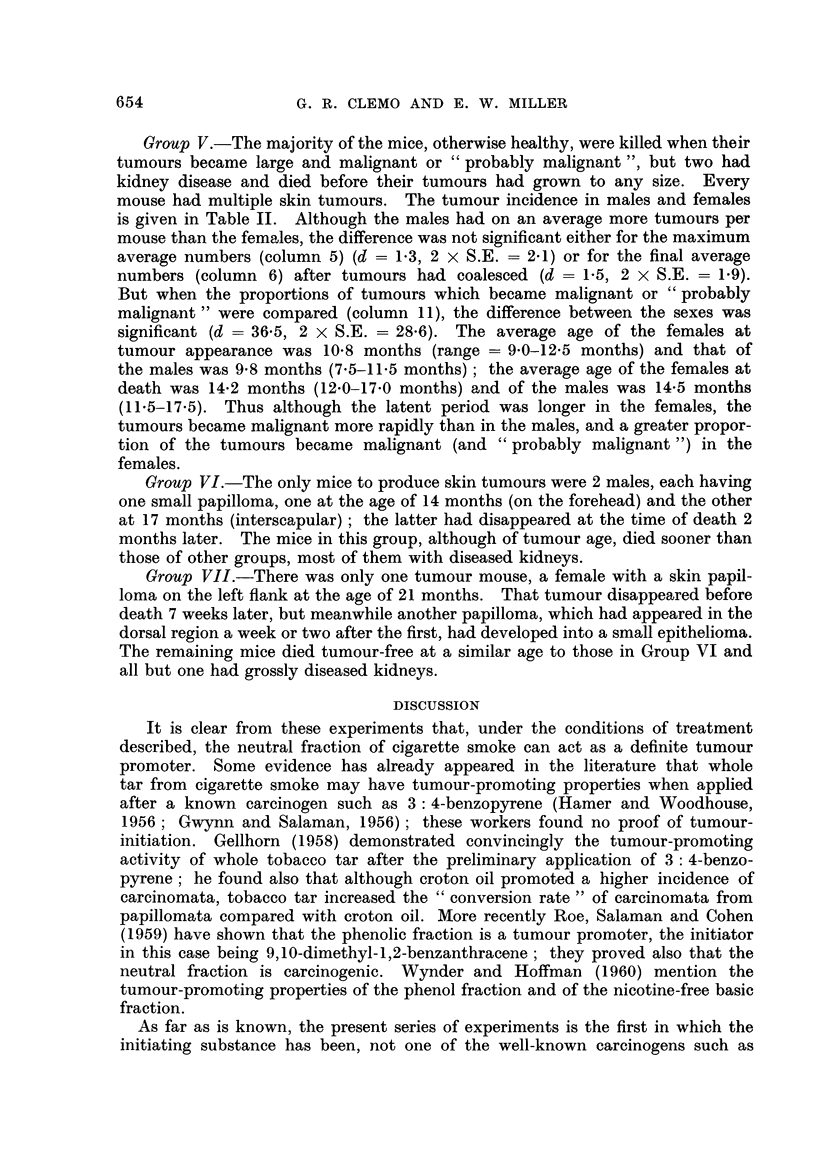

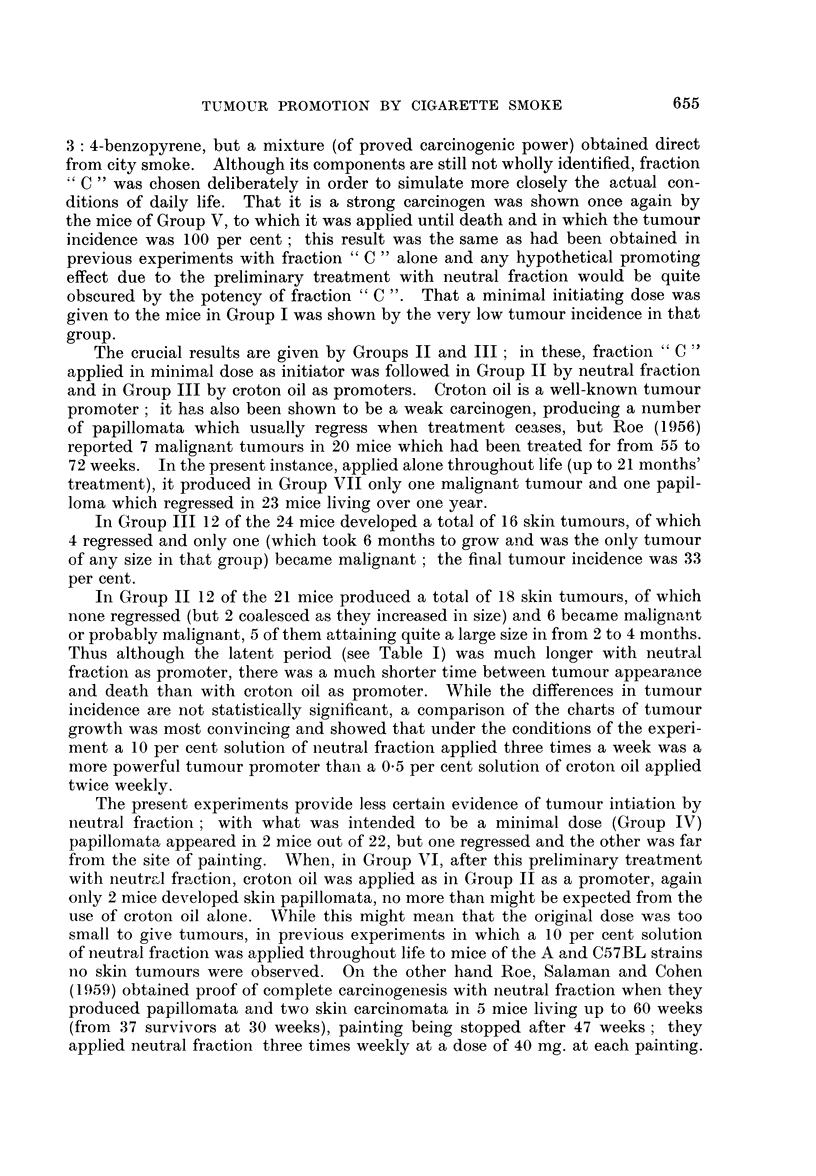

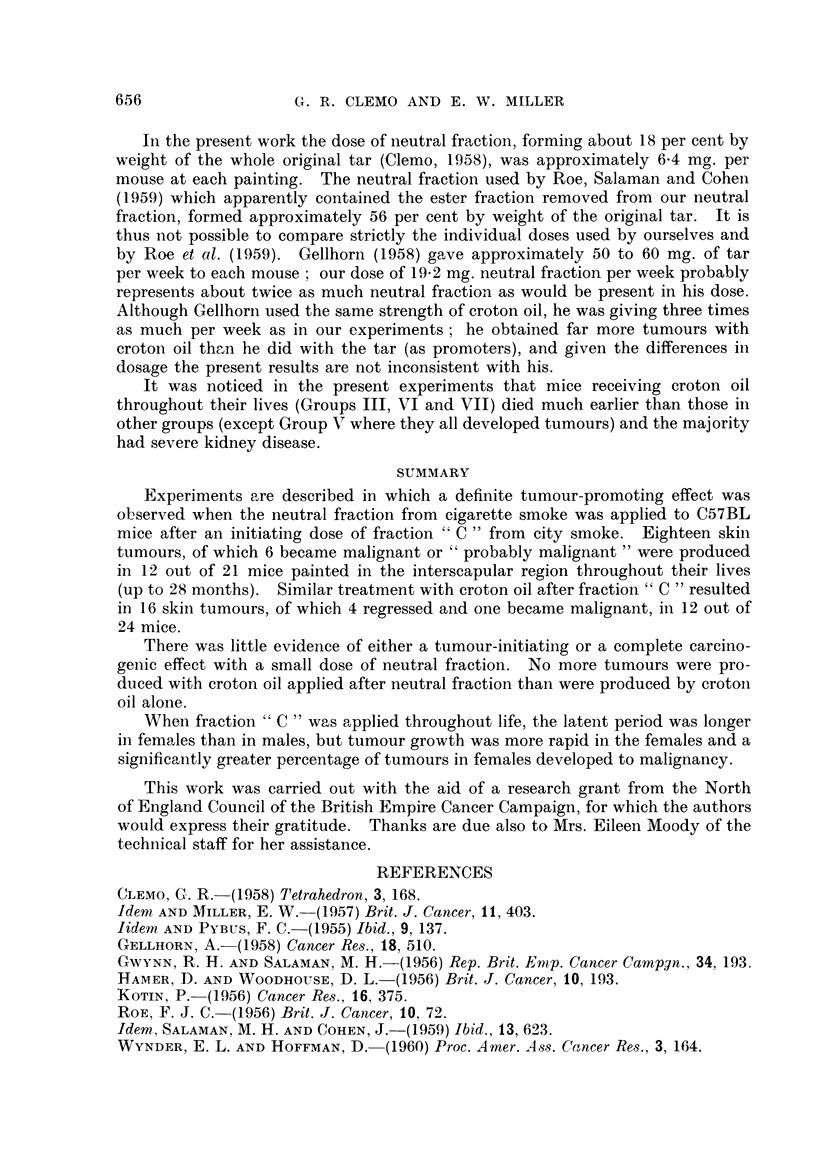

